# Photobiochemical mechanisms of biomolecules relevant to germicidal ultraviolet irradiation at 222 and 254 nm

**DOI:** 10.1038/s41598-022-22969-5

**Published:** 2022-10-29

**Authors:** Keisuke Naito, Kazuyuki Sawadaishi, Masahiro Kawasaki

**Affiliations:** 1 Light Source Business Division, Ushio Inc., Bessho, Himeji, 671-0224 Japan; 2 Carbuncle Bioscientech Inc., Yoshida-Kawaramachi, Kyoto, 606-8305 Japan; 3 Department of Molecular Engineering, Kyoto University, Kyoto, 615-8530 Japan; 4 Present Address: Arctic Research Center, Hokkaido University, Sapporo, 001-0021 Japan

**Keywords:** Biochemistry, Biological techniques, Biotechnology

## Abstract

To inactivate viruses and microorganisms, ultraviolet light in the short wavelength region is a promising candidate for mitigating the infection of disease. Germicidal mercury lamps emitting at 254 nm and KrCl excimer lamps emitting at 222 nm have sterilisation properties. In this work, wavelength dependence of the photobiochemical mechanisms was investigated with 222- and 254-nm irradiation to analyze the underlying damage mechanisms of DNA/RNA and proteins, using *Escherichia coli*, a protease, an oligopeptide, amino acids, plasmid DNA and nucleosides. The photorepair of damaged DNA and the “dark” reversion of the hydrates of uracil phosphoramidite coupling blocks were also investigated.

## Introduction

Ultraviolet (UV) light irradiation is an efficient way to inactivate viruses and microorganisms with the minimal undesirable effects on mammalian health or on the skin and eyes^1–5^. Previous studies have evaluated the wavelength pathogen response with the goal of ensuring that UV disinfection systems adequately protect human health, for example, from SARS-CoV-2^6–8^. An approach to prevent transmission of viruses is to inactivate airborne pathogens in public and transportation spaces, company offices, and hospitals when the spaces are occupied by people. This approach without harming the exposed mammalian skin can be achieved by short optical penetration depth of UV light. A low dose at 222 nm was efficient in inactivating aerosolised coronaviruses^6^. Irradiation at 222 nm on layered cell sheets was conducted, concluding that UV irradiation is biologically safe for cell viabilit^9,10^. An unfiltered broad spectrum 222-nm light was applied to control foodborne pathogens^11^. According to the literature of “a collection and analysis of a hundred years of data on results on the impact of UV irradiation on microorganisms, human and animal cells, skin and eyes”, the average necessary log-reduction doses at 222 nm are slightly higher compared to irradiation at 254 nm, and an appropriate dose should reduce most pathogens in most media by several orders of magnitude without harming human skin or eyes^12^.

UV irradiation induces damage to proteins and nucleic acids. Irradiation at 254 nm inactivated SARS-CoV-2 through the induction of viral genome damage and did not damage viral proteins^12^. Matrix- and nucleocapsid-proteins of viruses and microorganisms absorb UV light and reduce the density of light that reaches nucleic acids. Thus, at short UV wavelengths, the germicidal mechanism is mostly protein degradation, while at long UV wavelengths, nucleic acids are damaged^1,2,13–16^. Ribonucleic acid (RNA) and deoxyribonucleic acid (DNA) consist of a sugar-phosphate backbone protein and pyrimidine/purine bases. The UV action spectra for inducting cyclobutane pyrimidine dimers (CPDs) and pyrimidine (6-4)pyrimidone photoproducts ((6-4)PPs) in DNA peak at 260 nm and match the absorption spectrum of DNA dissolved in phosphate buffered saline, implying that direct photoabsorption by thymine induces DNA lesions^17^. Mechanistic insights into the photochemical formation of a hydrate adduct of the RNA nucleobase in an aqueous environment were reported^18^.

In this paper, we report the photochemical mechanisms of UV irradiation at 222 and 254 nm on biomolecules relevant to viruses and microorganisms; (a) degradation of aromatic amino acids, an oligopeptide, a protease and proteins, (b) degradation of plasmid DNA and its photorepair process after being transformed into *Escherichia coli* (*E. coli*) cells, (c) degradation of a cofactor in the CPD photorepair enzymatic process, (d) degradation of nucleosides, (e) product yields from RNA UpU and DNA dTpdT, and (f) self-reversion of the photohydrated UpU under dark conditions.

## Results

### UV damage of *Escherichia coli*

**Figure 1:**
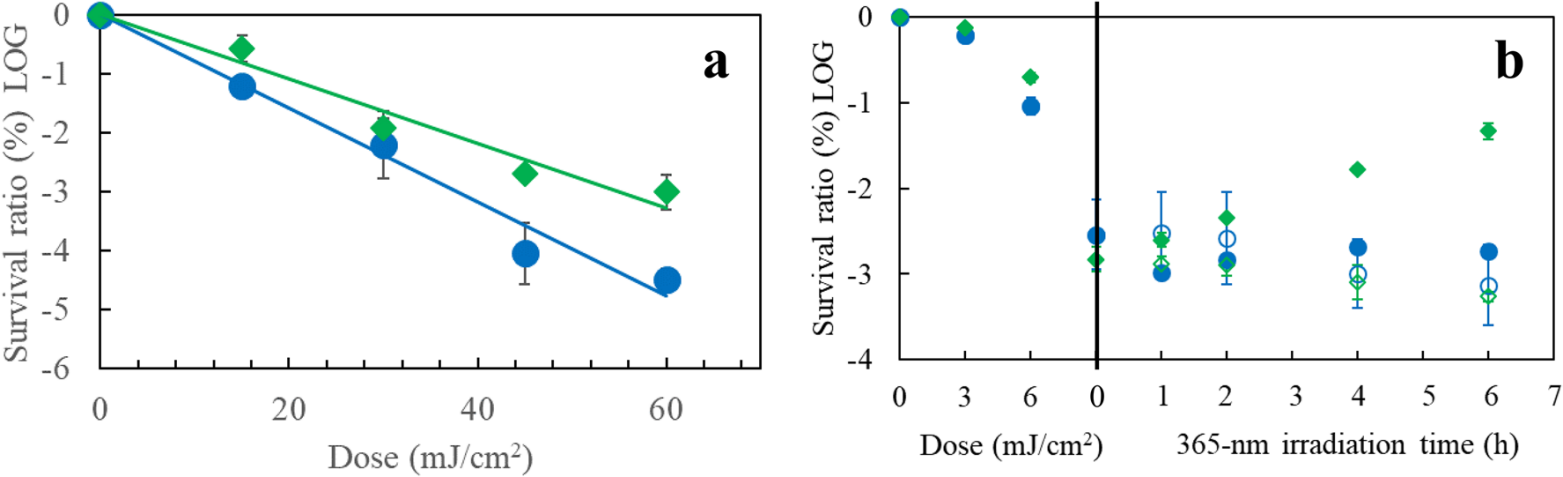
(**a**) Survival rates of *E. coli* bacterophage MS2 under UV irradiation. Log scale base 10. (Black circle) 222 nm, (Black diamond) 254 nm, The susceptibility ratio, *k*(222 nm)/*k*(254 nm) is 1.5 for the decays. *N* = 2 and *n* = 3, (**b**) (left half)survival rates of *E. coli* K-12 irradiated at 222 and 254 nm, (right half) photorepair rates by irirradiation at 365 nm. (Black circle) 222-nm irradiation followed by 365-nm irradiation, (white circle) 222-nm irradiation followed by preservation under dark conditions (control experiment), (black diamond) 254-nm irradiation followed by 365-nm irradiation, (white diamond) 254-nm irradiation followed by preservation under dark conditions (control experiment). *N* = *2 and n* = 3.

For survival measurements of *E. coli* bacteriophage MS2 cells, post UV irradiation, plaque counting of the cells cultured for 24 h on Luria–Bertani (LB) agar plates was performed as shown in Fig. 1a. The 222-nm irradiation resulted in 1.5-times faster decay than the 254-nm irradiation. Figure 1b shows the 365-nm (0.5 mJ/cm^2^) photorepair curves of *E. coli* K-12 cells after UV exposure to a dose of 9 mJ/cm^2^ at 222 and 254 nm. The cells irradiated at 222 nm did not recover, whereas those at 254 nm did. Post-photolysis “dark” repair process after UV exposure on *E. coli* K-12 at 222 and 254 nm was not observed during 6-h incubation.

**Figure 2:**
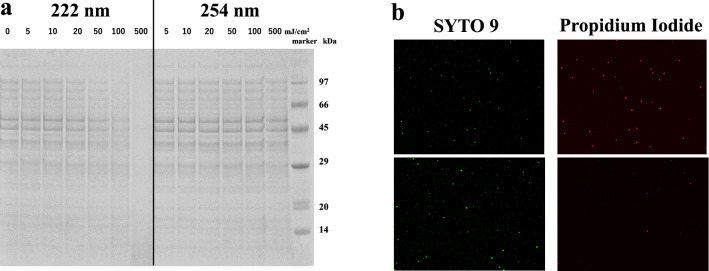
(**a**) SDS-PAGE of the *E. coli* K12 protein signature following exposure to increasing doses of UV light up to 500 mJ/cm^2^, as shown in each lane for 222- and 254-nm irradiation. (**b**) Fluorescent staining of *E. coli* K12 after UV irradiation. (upper) 222 nm and (lower) 254 nm with a dose of 200 mJ/cm^2^.

The image densitometry in Fig. 2a shows sodium dodecyl sulfate polyacrylamide gel electrophoresis with DTT added and without heating to detect signatures for proteins of *E. coli* K-12 (6.9-log CFU/mL). A dose of 50 mJ/cm^2^ at 222 nm reduced the densitometry intensity of the higher molar mass signatures, while no significant reduction was observed with a dose up to 500 mJ/cm^2^ at 254 nm. The similar wavelength dependence on bovine serum albumin was observed, as shown in Supplementary Fig. S1.

Cell membrane damage of *E. coli* K-12 was confirmed using the fluorescent staining method in Fig. 2b with a dose of 200 mJ/cm^2.^ Red fluorescence from propidium iodide was dominant in the sample irradiated at 222 nm, implying that the cells were dead with compromised cell membranes. The sample irradiated at 254 nm emitted green fluorescence from the SYTO9 dye in the cell membranes, implying that the cells had intact cell membranes.

### UV damage of a protease, an oligopeptide and amino acids

**Figure 3:**
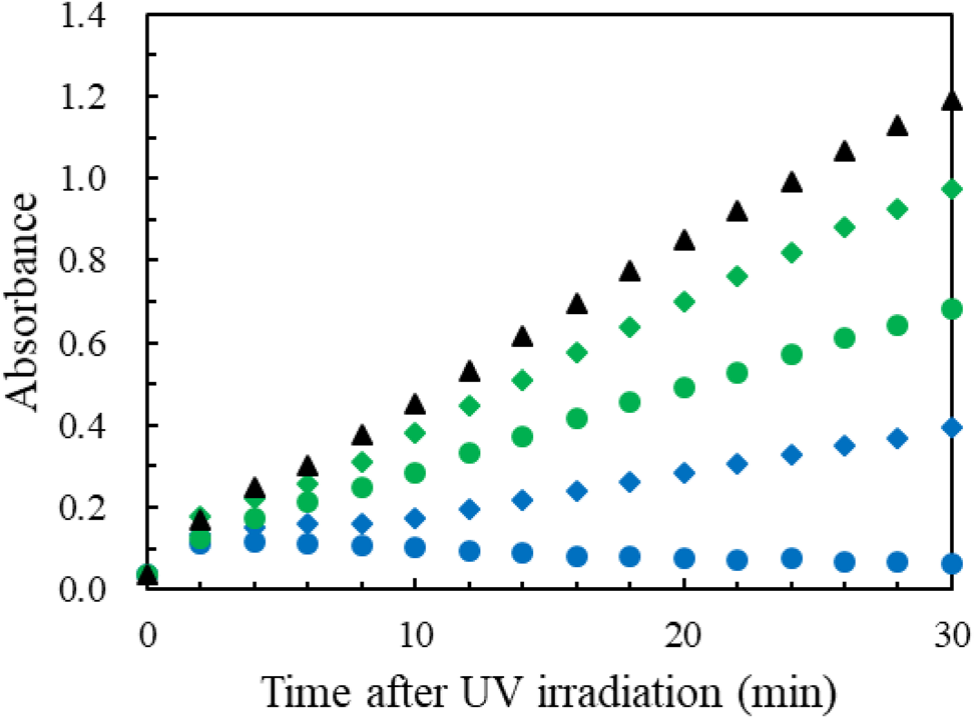
Production of *p*-nitroaniline by monitoring absorption intensity at 405 nm after UV irradiation to *aq.*solutions of chymotrypsin. UV doses in mJ/cm^2^ are (Blue diamond) 100 and (Blue circle) 500 at 222 nm, (green diamond) 100 and (green circle) 500 at 254 nm, (Black circle) 0. The initial hump is caused by light scattering. *n* = 1.

The absorption spectra of *aq.*solutions of the common aromatic amino acids, tryptophan (Trp), tyrosine (Tyr), phenylalanine (Phe) and histidine (His), are shown in Supplementary Fig. S2. His has absorption at 222 nm but not at 254 nm. Trp and Tyr have strong absorption at both 222 and 254 nm, whereas Phe has a weak absorption. A protease, chymotripsin, catalyses the peptide bond hydrolysis of Bz-Tyr-pNA (BTPNA in 50%DMSO / 50%water) in its S1 binding pocket of His 57, Ser 195 and Asp 102. To assess UV inhibition of the catalytic activity, *aq.* HCl solutions of α-chymotripsin were irradiated at 222 and 254 nm (0.5 mW/cm^2^) with doses of 100 and 500 mJ/cm^2^. After UV irradiation to α-chymotripsin, a mixture of the α-chymotripsin solution (75 μg/mL) and a BTNPA solution were monitored at a probe wavelength of 405 nm in a Tris∙HCl buffer solution, probing the hydration product, *p*-nitroaniline, as shown in Fig. 3. The higher dose and shorter UV wavelength resulted in less production of *p*-nitroaniline. The ratios of the slopes, *r* = (UV-irradiated)/(control), correspond to the residual catalytic activities. For a UV dose of 100 mJ/cm^2^, which is obtained from the data between* t* = 10–20 min in Fig. 3, *r*(222 nm) is 0.30, while *r*(254 nm) is 0.81 suggesting that the activity was 3.5 (= 70/19) times reduced by 222-nm irradiation than by 254-nm irradiation. The UV dose effects on the absorption spectra of the α-chymotripsin solutions were measured for does of 0–500 mJ/c^2^, as shown in Supplementary Fig. S3. The absorption spectra have a peak around 280 nm due to aromatic amino acid residues (Trp, Tyr and Phe). These chromophores absorb 254-nm photons to disorder the structure and lessen the catalytic ability without any appreciable change in its absorption spectrum. The spectral change after the 222-nm irradiation was proved at 225 nm (decrease of absorbance) and 250 nm (increase). The decreases in the catalytic ability and absorption spectral intensity imply the photodegradation of the His side chain in the binding pocket since (a) His not only stabilises developing charges, but also provides a path for proton transfer, without which catalytic reactions would have difficulty in proceeding, and (b) His is a strong chromophore at 222 nm.

Concerning the small reduction in the catalytic activity, *r*, after 254-nm irradiation, His is relevant to this because its weak photoabsorption. Although the catalytic reaction was inhibited, Supplementary Fig. S3 shows only a slight change in the UV absorption spectra after 254-nm irradiation. This is because the photoproducts might have a UV spectrum that resembles to the original α-chymotripsin one. McLaren and Luse^19^ reported that, in their 254-nm irradiation to chymotrypsin by chemical analysis, about one Trp residue per chymotrypsin molecule was destroyed and none of disulphide linkages were broken. No Phe, Tyr, or His residues were changed.

**Figure 4:**
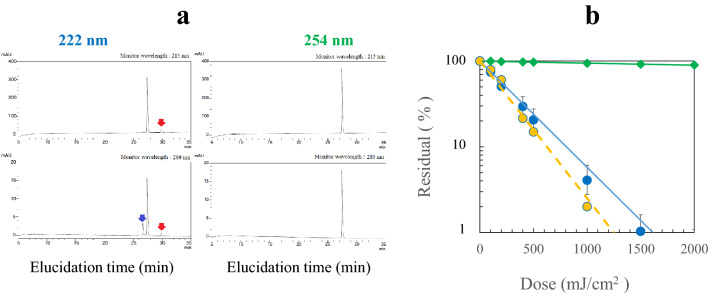
(**a**) HPLC elucidation profiles after UV irradiation of angiotensin II with a dose of 100 mJ/cm^2^ at 222 and at 254 nm. The monitoring wavelengths are (upper) 215 nm, and (lower) 280 nm. The red arrows indicate the photoproduct assingned to a peptide containing tyrosine, and the blue one to a peptide containing the product from photodegradation of histidine. (**b**) reduction of angiotensin II by UV irradiation. (Blue circle) 222 nm and (green diamond) 254 nm with *aq*. solutions aerated, (yellow circle) 222 nm with *aq*. solutions deaerated. *n* = 3.

To assess the role of the residues further, an oligopeptide, angiotensin II (Asp-Arg-Val-Tyr-Ile-His-Pro-Phe) in *aq.*solution (50 μM**)** was UV irradiated at 222 and 254 nm. The HPLC analysis for a dose of 100 mJ/cm^2^ is shown in Fig. 4a. For the 222-nm irradiation, the side peaks of the photoproduced peptides were observed at two monitor wavelengths of 215 and 280 nm, whereas no side peak for the 254-nm irradiation. The red arrows indicate the photoproduct assigned to a peptide containing Tyr since they appeared by monitoring at both 215 and 280 nm. The blue one to a peptide containing the products from the photodegradation of His since it appears strongly at 280 nm. Figure 4b shows that irradiation at 222 nm induced strong reduction, whereas it was very weak at 254 nm. Dose-dependent variations in the HPLC peak intensities are shown in Supplementary Fig. S4. For the dose of 0–1.0 J/cm^2^, the product intensity increased and the angiotensin II intensity decreased. Figure 4b also shows the deaeration effect of *aq*.solutions with N_2_ gas bubbling under sonication. Deaeration only slightly changed the reduction rate.

Figure 4b shows the wavelength dependence of the reduction of angiotensin II with strong reduction by irradiation at 222 nm and almost no reduction 254 nm. To assess the photodegradation role of the amino acid residues, free Tyr, Trp, Phe and His, *aq*. solutions (50 μM) were irradiated at 222 and 254 nm for doses of 0– 2 J/cm^2^ in Supplementary Fig. S5. The residual percentages at a dose of 1.0 J/cm^2^ were as follows: at 222 nm His (< 1%): Trp (16%): Tyr (38%): Phe (82%), and at 254 nm His (100%):Trp (71%): Tyr (88%): Phe (95%). Tyr is appreciably reduced at 254 nm and ca. eightfold less reduced than His at 222 nm. Thus, the photoreduction susceptibility to His matches the wavelength dependence of the observed reduction rates of angiotensin II in Fig. 4b, while not to Tyr, since free Tyr has appreciable absorption at 245 nm and His has no absorption. The deaeration effect of the angiotensin II solution is small in Fig. 4b. In the oxidation process of free aromatic amino acids, it has been kown that His and Trp react at appreciable rates with singlet oxygen^20^. As shown in Supplementary Fig. S5, the deaeration twofold decreased the UV reduction rate for Trp during 222-nm irradiation, but not for His. Thus, His residue is the most plausible for the UV reduction of angiotensin II. The deaeration of *aq*. solution did not afford protection against His degradation at 222 nm, implying that the primary photochemistry at 222 nm is direct photodegradation and independent of dissolved O_2_.

### UV damage of plasmid DNA at 222 and 254 nm and photorepair ability at 365 nm

**Figure 5:**
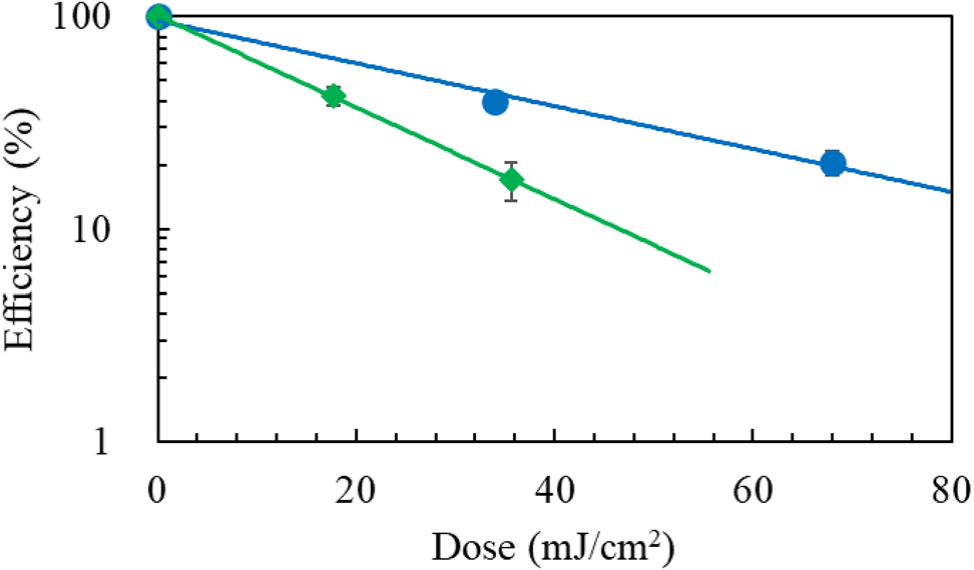
Transformation efficiencis of *E. coli* HB101 as a function of UV dose to plasmid DNA. (Blue circle) 222 nm and (green diamond) 254 nm. *n* = 1.

Following UV excitation on DNA, adjacent pyrimidines may form lesions, CPD or (6-4)PP. The CPD lesion is photorepaired by a photolyase and a coenzyme (flavin adenine dinucleotide, FAD) by long-wavelength light, while the (6-4)PP lesion is not. We examined UV damage of plasmid DNA (1, 5, 10 pg) in Tris∙EDTA buffer solutions at 222 and 254 nm, which were transformed into *E. coli* HB101 competent cells after UV irradiation. Figure 5 shows the transformation efficiency curves, for which the raw data of colony counting are listed in Supplementary Table S1. The doses at 21 and 40% transformation were 68 and 34 mJ/cm^2^ for 222 nm, while for 254 nm the doses at 17 and 42% transformation were 36 and 18 mJ/cm^2^ The photodamage susceptibility of plasmid DNA is twofold lower at 222 nm than at 254 nm because of the weaker absorbance of DNA at 222 nm^14^.

To assess the photorepair ability of the damaged DNA, two sets of plasmid DNA samples that were reduced to 20% residual by UV irradiation were transformed into *E. coli* in ampicillin LB plates. After 60 min with and without photorepair irradiation at 365 nm (0.36 mW/cm^2^), the plates were placed for cultivation, and then, colony counting was performed. The average colony numbers for the 222-nm irradiation were *N*_222_ (with 365 nm) = 61 and *N*_222_ (without 365 nm) = 40. For the 254-nm irradiation, *N*_254_ (with 365 nm) = 61 and *N*_254_ (without 365 nm) = 31. The photorepair increment ratio at 222 nm is *R*_222_ = *N*_222_ (with 365 nm)/*N*_222_(without 365 nm)—1 = 61/40–1 = 0.53, while at 254 nm, *R*_254_ = 61/31–1 = 0.97. These results imply that (a) production of CPD in plasmid DNA irradiated at 222 nm is less than at 254 nm and/or (b) CPD photorepair by the photolyase is less active at 222 nm than at 254 nm.

### Photodamage of a cofactor FAD in the CPD photorepair enzymatic process

**Figure 6:**
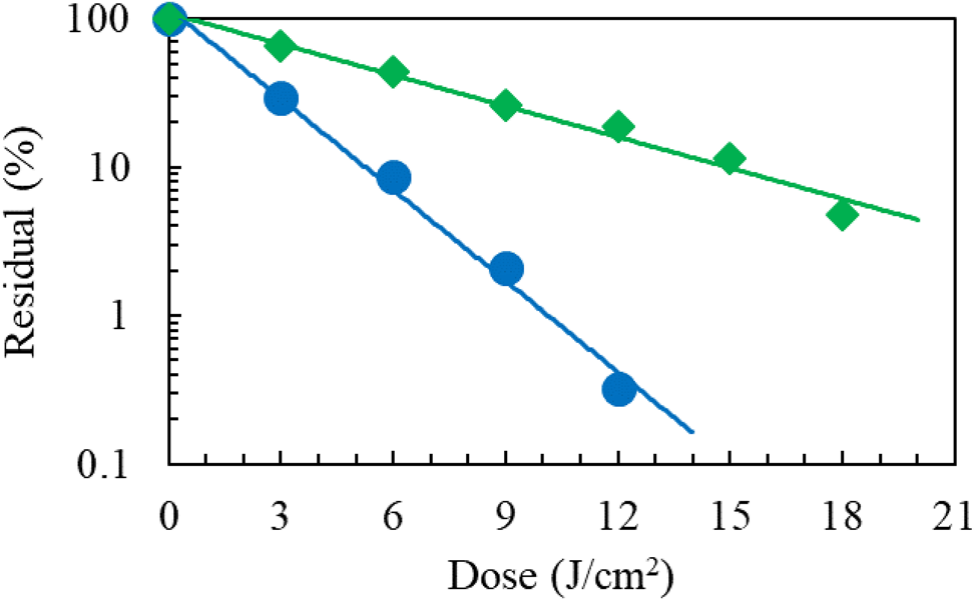
Reduction of FAD as a function of dose. UV irradiation at (blue circle) 222 nm and (green diamond) 254 nm.* n* = 1.

The photolyase harbours an FAD coenzyme to reverse CPD to the adjacent pyrimidines. As shown in Fig. 6, UV irradiation of FAD (25 μg/mL) in *aq.* solution at 222 nm up to a dose of 18 J/cm^2^ induced damage three-fold more efficiently than at 254 nm, implying that CPD photorepairs by the photolyase became less active by irradiation at 222 nm than at 254 nm. HPLC elucidation profiles are shown in Supplementary Fig. S6.

### UV damage of nucleosides

**Figure 7:**
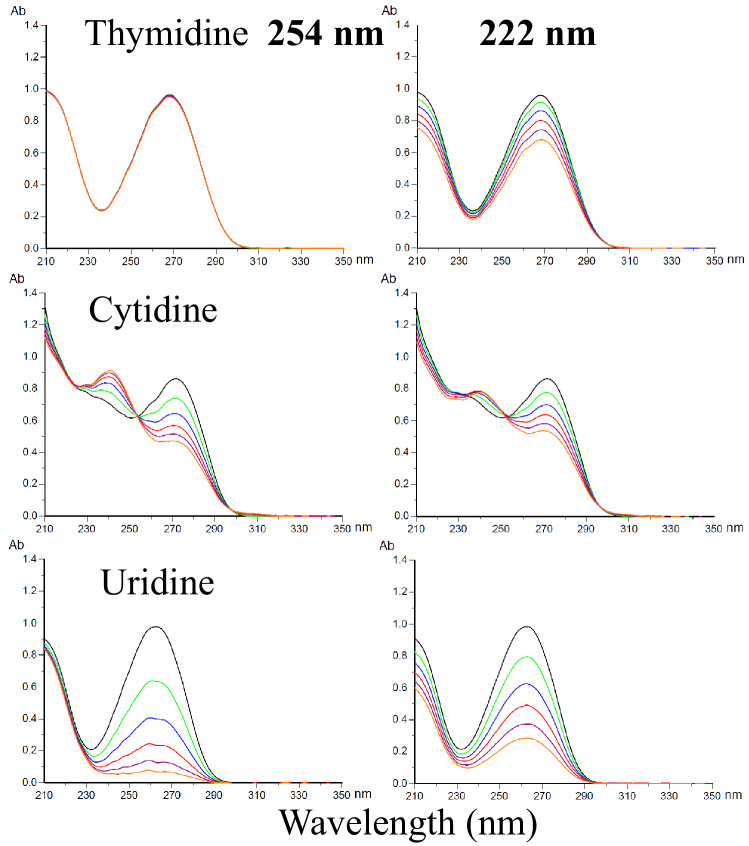
Changes in the absorption spectra of pyrimidine nucleosides following exposure to increasing UV dose at 222 nm and 254 nm up to 5.0 J/cm^2^ with a step 1.0 J/cm^2^ from the spectrum in black to one in orange. Concetration of *aq*.solutions = 50 μM and optical path length = 10 mm.

DNA consists of a sugar-phosphate backbone to which the four nucleobases, adenine (A), guanine (G), cytosine (C), thymine (T), are attached by glycosidic bonds, while T is replaced by uracil (U) in RNA. The absorption spectra of nucleosides have a peak in the range 250–270 nm, and the backbone absorbs at around 210 nm. The photodamage of these nucleosides in *aq.* solution (50 μM) was investigated by measurement of absorption spectral changes. As shown in Fig. 7 with doses up to 5.0 J/cm^2^, the pyrimidine bases, C and U, were comparably photodamaged by 222-nm and 254-nm irradiation. The pyrimidine base, T, was photodamaged at 222 nm but not at 254 nm. Thus, T is most plausible to the UV damage of plasmid DNA at 222 nm in Fig. 5. Similarly, no appreciable changes in the absorption spectrum of the purine nucleobases, adenosine and guanosine, were observed upon UV irradiation, as shown in Supplementary Fig. S7. Since the mechanism of photo-damage is largely different between nucleoside monomer and plasmid due to their environment difference, additional experiments are needed to confirm these assumptions.

### UV damage of RNA UpU

**Figure 8:**
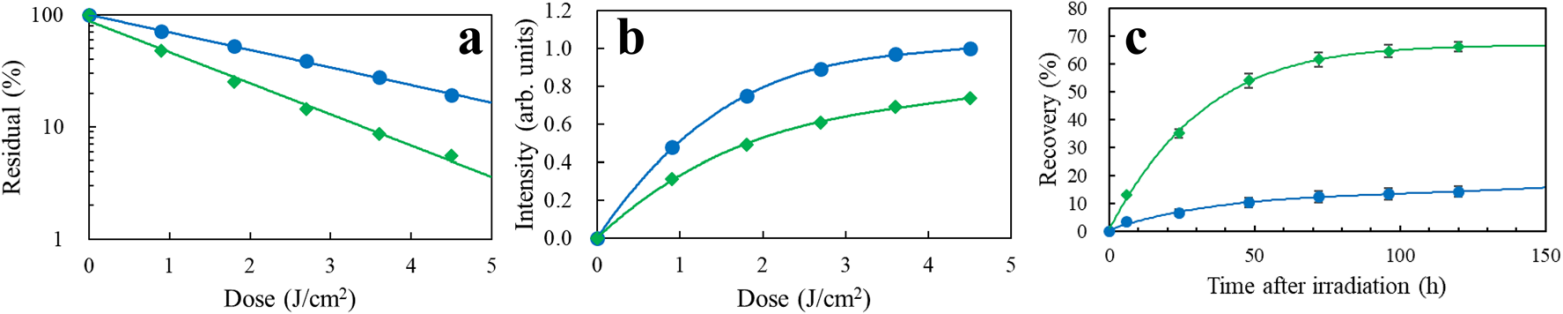
UV photochemistry of RNA UpU. (Blue circle) 222 nm and (green diamond) 254 nm (**a**) reduction of UpU as a function of dose. *n* = 1, (**b**) production of (6-4)PP from UpU as a function of dose. *n* = 1, (**c**) recovery of photohydrated UpU due to the self-reversion reaction at room temperature under dark conditions after UV irradiation. SD = 2% of each signal intensity. *n* = 3.

RNA UpU is a model nucleotide block for RNA. UpU (50 μg/mL) in *aq.*solution was UV irradiated to assess the wavelength sensitivities to damage of UpU and formation of (6-4)PP and CPD. By HPLC analysis with monitoring at 258 nm, the reduction of UpU by irradiation at 222 and 254 nm up to a dose of 4.5 J/cm^2^ was measured, as shown in Fig. 8a. The degradation rate at 222 nm was 1.9 ± 0.1 times lower than that at 254 nm. Figure 8b shows that the production of (6-4)PP from UpU is 1.5-times larger by irradiation at 222 nm than at 254 nm, implying that the production of (6-4)PP at 222 nm is 2.9-time (1.9 × 1.5) more efficient than at 254 nm. Supplementary Figs. S8 and S9 show the spectral changes of UpU by UV irradiation and HPLC elucidation profiles of the products, respectively. The HPLC analyses show that a weak CPD signal was observed for 222-nm irradiation, whereas a strong one for 254-nm irradiation. Based on these results, the photorepair yield of RNA damaged by irradiation at 222 nm is expected to be low in a germicidal process.

UV irradiation of dTpdT produced only (6-4)PP at 222 nm, while both (6-4)PP and CPD at 254 nm, as shown in the HPLC elucidation profiles of Supplementary Fig. S10.

### Production of hydrate from UpU and its “dark” reversion

In addition to ordinary photodamage processes by UV irradiation, a water molecule adds across the pre-existing double bond in a nucleophilic hydrolysis reaction and is equilibrated with the non-hydrated UpU^18^. The reversible reaction of hydrated UpU was examined with these samples subsequently preserved for up to 120 h at room temperature under dark conditions. The self-reversion process from hydrated UpU to non-hydrated one was monitored at 215-nm absorption of UpU in HPLC analysis. The elucidation profiles after 254-nm irradiation are shown in Supplementary Fig. S11. Figure 8c shows the self-recovery curves of UpU damaged by UV irradiation. UpU was damaged to a 4% residual (96% damage) by irradiation at 254 nm at a dose of 4.5 J/cm^2^. After preservation for 120 h, UpU recovered to a 68% residual. Thus, the recovery amount was 64%. At 222 nm with a dose of 7.2 J/cm^2^ UpU was damaged to a 11% residual (89% damage), which recovered to a 24% residual. Thus, the recovery amount was 13%.

Note that the formation of thymine hydrates by 254-nm irradiation has a low quantum yield due to steric hindrance by the 5-CH_3_ substituent in thymine. As shown in the HPLC elucidation profiles in Supplementary Fig. S10, UV irradiation of dTpdT at 254 nm produced CPD and (6-4)PP, whereas only (6-4)PP at 222 nm.

Table 1 summarizes the comparison of damages and products caused by 222-nm and 254-nm irradiation on cell proteins, chymotripsin, angiotensin II, plasmid DNA, FAD photorepair coenzyme and uracil/thymine dimer blocks.

**Table 1:** Comparison of damages and products caused by 222- and 254-nm irradiation.

Wavelength (nm)	*E. coli* protein	Plasmid DNA^a^		FAD	UpU^b^		dTpdT^c^	Angiotensin II	Chymotripsin
	Damage	Damage	Photorepair	Damage	Damage	Product	Product	Damage	Catalyst ability
222	High	Low	Low	High	Low	(6-4)PP/hydrate	(6-4)PP	High	High reduction
254	Low	High	High	Low	High	CPD/ (6-4)PP/hydrate	CPD/ (6-4)PP	Low	Low reduction

FAD, flavin adenine dinucleotide; Angiotensin II, an oligopeptide of Asp-Arg-Val-Tyr-Ile-His-Pro-Phe; Chymotripsin, a protease with a binding pocket of His, Ser and Asp; (6-4)PP, (6-4)photoproduct of pyrimidine dimer; CPD, cyclobutane pyrimidine dimer.

^a^After plasmid DNA is irradiated by UV light, it is transformed into an *E. coli* cell.

^b^RNA UpU.

^c^DNA dTpdT.

## Discussion

Viruses and microorganisms consist of proteins, nucleic acids and carbohydrates, and their photochemistry is governed by photoabsorption properties. The weak absorbance of proteins at 254 − 280 nm is dominated by amino acids, and the tenfold strong absorption around 200 nm by the peptide bond^2,21^. UV absorption of proteins exceeds that of DNA at 220 nm, and its minimum is at 250 nm. Genomic damage of adenovirus was induced by irradiation at 254 nm, while its protein damage at 222 nm^15,16^. The 220-nm irradiation damaged its hexon and penton proteins^22^. Since the partial gene analysis of its hexon protein revealed 22 aromatic amino acids (17 Tyr, 2 Trp and 3 His) among 242 amino acids^23^, UV irradiation may induce direct photodamage of the amino acid residues. In the present study, the observed wavelength dependence of the UV degradation of chymotrypsin and angiotensin II at 222 nm implies a direct photoprocess of His residues. The photodamage susceptibility of His at 222 nm is highest among the aromatic amino acids, which is due to the difference in the photostability between the imidazole and phenyl groups. Immunoblotting analysis to viral spike and nucleocapsid proteins of SARS-CoV-2 showed that 254-nm irradiation did not induce damage of viral proteins^13^, which is consistent with the present wavelength dependence of the His degradation. The 237-nm irradiation on monoclonal antibodies oxidised His in modification products of individual His residues to Asp (and/or iso-Asp) and Asn^24^. However, the oxidation reaction of free His was slow in the present study of 222-nm irradiation. This wavelength dependence in the UV-C photochemistry of His may be explained by the fact that there are two molecular electronic excited states closely located in these wavelengths, that is, the 220 nm band is mainly assigned to π-π* excitation and the n-π* state is partly populated at the longer wavelength^25^.

The viral infectivity spectrum matches the RNA absorption spectrum in the long wavelength region, while it does the protein spectrum in the short wavelngth^26,27^. UV irradiation below 240 nm damages viral proteins^15,22^. UV disinfection of *E. coli* bacteriophage MS2 is less enhanced by any nongenomic mechanism at below 240 nm, contrary to adenovirus disinfection. This difference is most likely due to differences in their viral proteins. In Fig. 1, the germicidal effects for *E. coli* cells at 222 nm are stronger than those at 254 nm, while in Fig. 5 the wavelength effect for plasmid DNA is in the reverse order. These results support the efficient protein damage process in viruses and microorganisms by 222-nm irradiation.

After exposure at 220–300 nm with a medium-pressure mercury lamp, almost no photorepair of *E. coli* K-12 was reported due to the disorder of the photolyase^28^. Photodegradation of proteins and inactivation of enzymes were much more effective by 222-nm irradiation compared to 254 nm^29^. Thus, the low photorepair yield of plasmid DNA damaged at 222 nm in the present work is due to UV degradation of photolyase/FAD as well as the low yield of CPD lesions.

Concerning self-reversion of photoinduced damage in UpU, it was reported that uridine photohydrates were formed following UV irradiation of single-stranded R17-RNA via a photoinduced nucleophilic hydrolysis reaction, in which water is split into H atom and OH radical via photosensitisation of uracil^30^. These nascent radicals then add across the pre-existing double bond to form the hydrates. The nascent hydrate adduct was stable at 37 °C in a neutral aqueous solution, which was in equilibrium with the non-hydrated RNA. In our experiments for UpU hydration, the self-reversion yield to unhydrated UpU during preservation was about fourfold lower for UpU irradiated at 222 nm than at 254 nm. This might increase the germicidal efficacy of 222-nm irradiation. To evaluate the germicidal efficacy at 254 nm for viruses, this reversion process should be considered. For example, survival measurements of *E. coli* bacteriophage MS2 cells in Fig. 1a, a substantial amount of the hydrated uracils self-revcovered to non-hydrated ones after 24 h cultivation process in the 254-nm irradiation experiment, but a much less amount in the 222-nm irradiation experiment.

A theoretical calculation for the hydration mapped the reaction paths associated with the reactivity of U with H_2_O^18^. The vertical excitation energies associated with the lowest two electronically excited states of the U + H_2_O complex are the electronically excited states, S_1_ at 4.96 eV and S_2_ at 5.20 eV above the ground S_0_ state. The reaction path via a minimum energy is a conical intersection for forming a 6-HU hydrate adduct. At near-threshold excitation to S_1_, such couplings would need to surmount an energy barrier of 5 eV associated with orthogonal nuclear motions to alternative reactions. The UV photon energies at 254 nm (4.9 eV) and 222 nm (5.6 eV) are close, but may induce different intersection paths via S_1_ and S_2_, respectively. Thus, the branching ratio of hydrate production depends on the UV wavelength.

## Materials and methods

### Fluorescent staining methods for protein bactericidal mechanism study

To assess cell membrane damage in *E. coli* NBRC.106373 cells in LB broth, a commercial kit (LIVE/DEAD *Bac*Light Bacterial Viability Kit, Thermo Fisher Scientific, Waltham, MA, USA) was used as a function of membrane integrity. After incubation and centrifugal washing, the *E. coli* cells in PBS were irradiated at 222 and 254 nm. The UV intensity and dose were 0.1 mW/cm^2^ and 200 mJ/cm^2^, respectively. The sample was deployed on an agar medium and incubated at 37 °C for 48 h for survival curve measurement. The sample was also stained with a mixture of SYTO9 dye, and propidium iodide provided in the kit. Fluorescence from live or dead *E. coli* K-12 cells was measured at an excitation wavelength of 485 nm. The bacteria with intact cell membranes fluoresce green from SYTO9 in the cell membranes, while dead cells with compromised membranes fluoresce red because propidium iodide emits red fluorescence when binding to nucleic acids and does not pass through intact cell membranes.

### UV degradation of plasmid DNA transformed into *E. coli*

For UV damage tests of plasmid DNA pBR322 (Takara Bio, Kusatsu, Japan) by irradiation at 222 and 254 nm, *E. coli* HB101 competent cells Quick DH5α (DNA-913) were purchased from TOYOBO (Osaka, Japan). Upon UV exposure on DNA 0.1 μg/mL in TE buffer solutions (pH7.5, 1 mM Tris–HCl/0.1 mM EDTA), *E. coli* HB101 cells were transformed with the plasmid DNA and cultured on LB agar plates containing ampicillin (50 μg/mL, Nacalai Tesque, Kyoto, Japan).

### Preparation of reactants, analysis of photoproducts and properties of lamps

RNA UpU was prepared, in which a levulinyl group was chosen for transient protection of the 3′-hydroxyl group. Synthesis products were analyzed using reversed-phase HPLC and UV absorption spectrometry as described in Supplementary Information. In the protease experiment, we prepared (1) solution A: α-chymotripsin (75 μg/mL, Nacalai Tesque, Kyoto, Japan) in *aq.*solution (HCl 1 mM), (2) solution B: BTNPA (1.6 mg/mL, Peptide Lab. Ibaraki, Japan) in 50% DMSO aq.solution and C) buffer solution C: 100 mM Tris∙HCl pH8. After solution A was UV irradiated at 30 °C, to assess UV inhibition of the catalytic activity, a mixture of solution A(60 cc), solution B(30 cc), solution C(150 cc) and water (60 cc) was monitored at a wavelength of 405 nm, probing the hydration product, *p*-nitroaniline. In absorption spectral measurements, the dilution effect caused by the reaction-stopping reagent was taken into accounts. An oligopeptide, angiotensin II (human)∙AcOH∙ 4H_2_O was purchased from Peptide Lab. (Code 4001, Ibaraki, Japan). The HPLC instrument for analysis of synthesized samples and photoproducts was Shimadzu Prominence with a column of TSKgel ODS-80Ts (Tosoh, Tokyo, Japan) and solutions of triethylamine acetate (TEAA) and TEAA/acetonitrile. FAD and bovine serum albumin (Nacalai Tesque, Kyoto, Japan) were used without further purification. FAD was analysed with a 4:1 (potassuim dhydrogen phosphate: methanol) solution. dTpdT was purchased from TriLink Biotechnologies (San Diego, USA). Amino acids (Trp, Tyr, Phe, His) in *aq*. solutions (Nacalai Tesque) were measured by HPLC with *aq*. solution of acetonitrile/trifluoroacetic acid (TFA) for Trp, Tyr and Phe, and sodium 1-pentanesulfonic acid/acetnitrile/phosphoric acid for His. Photoproducts from UV irradiated angiotensin II were measured with solutions of TFA and *aq.* TFA/acetonitrile. Nucleosides (A, G, C, T, U) were purchased from Tokyo Kasei (Tokyo, Japan). Ultraviolet germicidal irradiation disinfection treatments used UV light emitted from a filtered narrow spectrum 222-nm KrCl light source (222 nm, 100 μW/cm^2^ at 293 mm distance, Ushio Inc., Tokyo, Japan with 235–280 nm light removed) or a broad spectrum 254-nm low-pressure mercury lamp (254 nm, 100 μW/cm2 at 330 mm distance). The VUV spectrum was reported in Ref. 5 and a UV meter was described therein.

A description of the correction of the UV dose to samples for photoabsorption by media solutions and culture plates is provided in Supplementary Information. We refer to the effective dose in this analysis. Deaeration of water was performed by bubbling of nitrogen gas with ultrasonification. Oxygen concentrations were 8 and 0.1 mg/L before and after the deaeration procedure, respectively. Replication numbers, *N* (biological), are three for all measurements unless otherwise stated, and *n* (technical) were variable, which are described in the figure legends. An experiment was not replicated when the difference between the results at 222- and 254-nm irradiation was clear.

## Supplementary Information

Supplementary Information.

## Data Availability

The datasets supporting the conclusions of this article are included in the article and Supplementary Information. If readers would like further information about the data, please contact K.N.
