# Supplementary material for: Photobiochemical mechanisms of biomolecules relevant to germicidal ultraviolet irradiation at 222 and 254 nm
Source: Sci Rep. 2022 Oct 29;12:18217. doi: 10.1038/s41598-022-22969-5 (PMC9617911; doi:10.1038/s41598-022-22969-5)
Supplement: Supplementary file 1 — Supplementary Information. [file 41598_2022_22969_MOESM1_ESM.pdf]

Supplementary Information

Photobiochemical mechanisms of biomolecules relevant to germicidal ultraviolet irradiation at 222 and 254 nm

Keisuke Naito, Kazuyuki Sawadaishi & Masahiro Kawasaki

1. Gel electrophoresis for albumin after UV irradiation at 222 nm and 254 nm.

SDS-PAGE (Fig. S1) shows the bovin serum albumin (Sigma Aldrich) signature at 66 kDa across the germicidal UV spectrum, following exposure to increasing doses of UV light up to 500 mJ/cm^2 in each lane of 222 and 254 nm. For this analysis, no heating process was applied. Proteins were identified using the molecular mass labelled on the side bar. At a high dose of 222-nm irradiation, diffused signals were observed in the low molecular weight region.

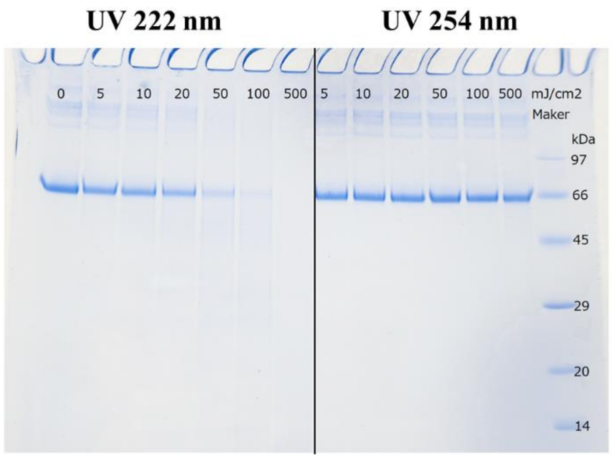

Fig. S1 SDS-PAGE of the bovin serum albumin after exposure to 222-nm irradiation (left) and 254-nm irradiation (right)

2. Absorption spectra of aromatic amino acids

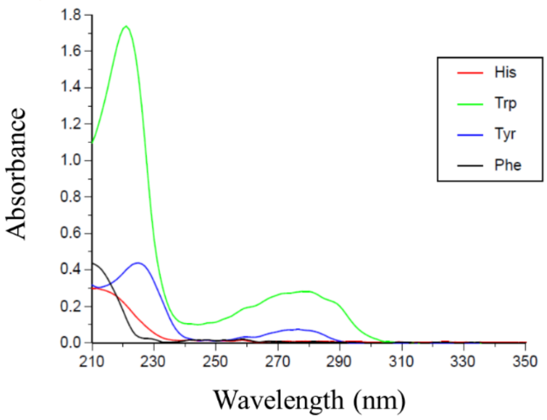

Fig. S2 UV absorption spectra of the aromatic amino acids, tryptophan (Trp), tyrosine (Tyr), phenyalanine (Phe) and histidine (His) in *aq*. solutions (50 µM). Optical path length = 10 mm

3. Absorption spectral change of chymotripsin solutions after UV irradiation

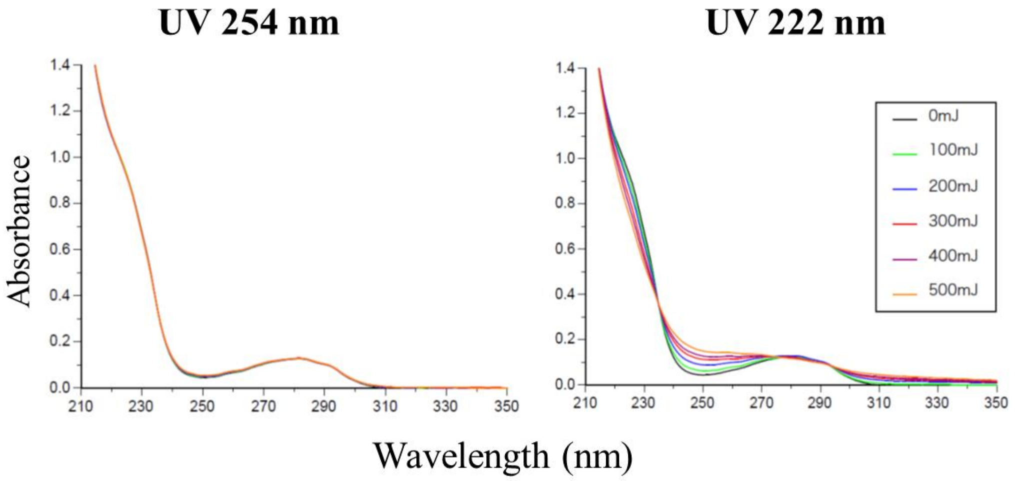

Fig. S3 Absorption spectra of α-chymotripsin solutions (75 μg/mL). UV
[illegible]

4. UV degradation of angiotensin II (Asp-Arg-Val-Tyr-Ile-His-Pro-Phe) at 222 nm

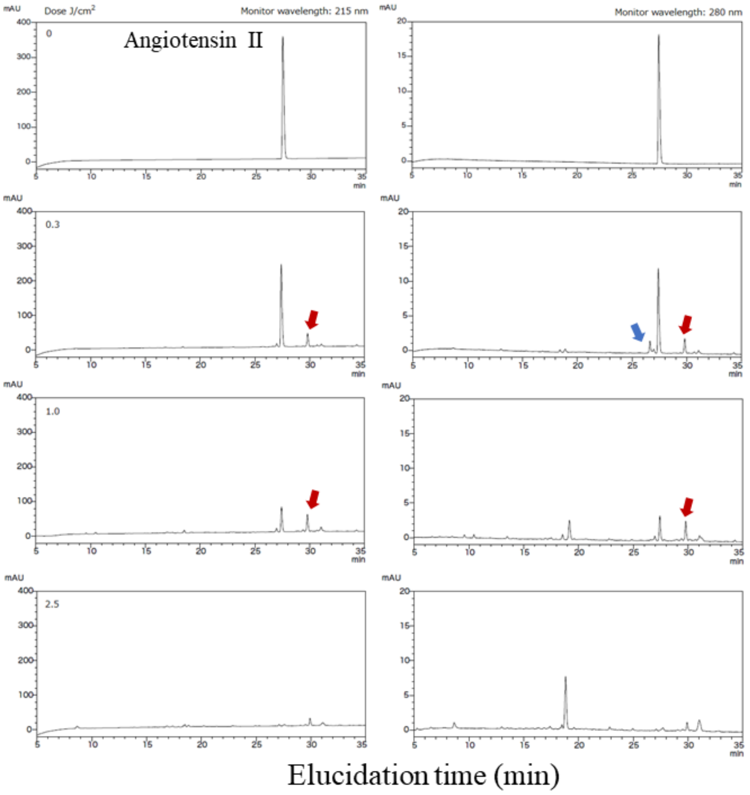

Fig. S4 HPLC elucidation profiles (up to 20 min) of angiotensin II after 222-nm irradiation. Monitor wavelengths are (**left**) 215 nm and (**right**) 280 nm. UV doses from the top are 0, 0.3, 1.0 and 2.5 J/cm^2. The red arrows indicate the photoproduct assigned to a peptide containing tyrosine, and the blue one to a peptide containing the product from photodegradation of histidine.

5. Reduction curves of aromatic amino acids in aerated and deaerated *aq.*solutions

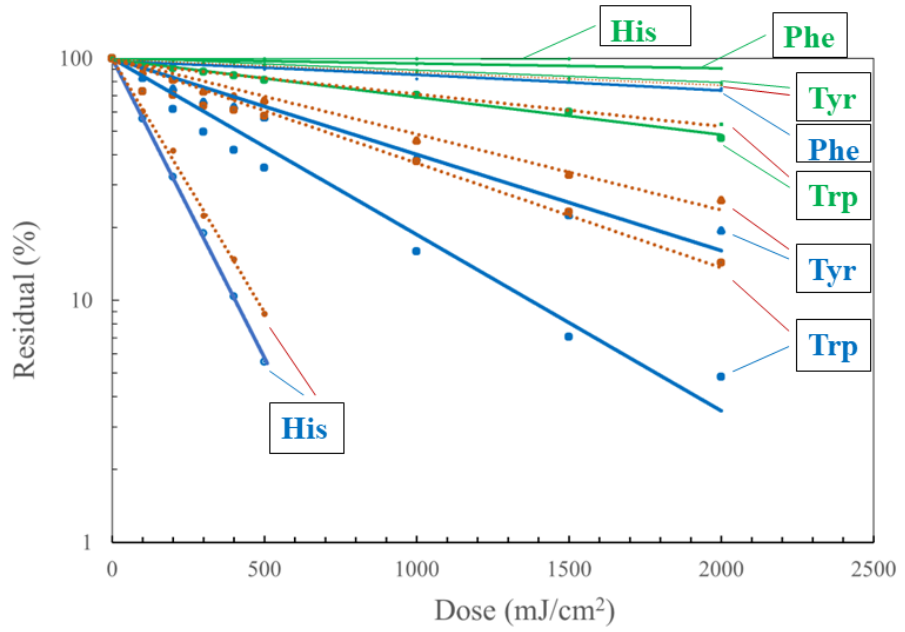

Fig. S5 Reduction of (●) His, (■) Trp, (▲) Tyr and (♦) Phe in *aq*. solutions (50 µM) by UV irradiation at (**blue)** 222 nm and (**green**) 254 nm. (**khaki**) in deaerated *aq*. solutions. The residual amounts of each amino acid were analysed with HPLC.

6. Colony formation of *E. coli* transformed with plasmid DNA photodamaged by irradiation at 222 and 254 nm

Plasmid DNA solutions were irradiated at 222 and 254 nm to examine the DNA damage. After UV irradiation, photodamaged DNA was transferred into *E. coli* HB101 competent cells that were cultivated for colony counting. UV intensity was 0.5 mW/cm^2.

Table S1 Transformation of *E. coli* HB101 competent cells by UV-irradiated plasmid DNA

UV	Dose mJ/cm^2	DNA pg	colony count # 1	#2	#3	avr.	count/DNA pg	count/DNA pg avr.	transformation %	damage %
–	0	5	430	488	512	477	95			
		1	103	112	113	109	109	102	100	0
222 nm	34	5	202	205	182	196	39			
		1	37	41	49	42	42	41	40	60
	68	10	196	228	261	228	23			
		5	108	108	120	112	22			
		1	18	18	18	18	18	21	21	79
254 nm	18	5	188	204	244	212	42			
		1	42	42	49	44	44	43	42	58
	36	10	137	179	191	169	17			
		5	74	57	95	75	15			
		1	18	18	25	20	20	17	17	83

7. Photodegradation of FAD by 222-nm and 254-nm irradiation

HPLC analyses of the photoproducts of FAD at 222 and 254 nm were performed using triethylamine acetate (TEAA) buffer solution (pH = 7.0) and TEAA/acetonitrile (pH7.0) at the monitoring wavelengths of **(upper panel)** 450 nm and (**lower panel)** 254 nm in Fig. S6. Choromophors are adenosine and riboflavin in FAD, respectively.

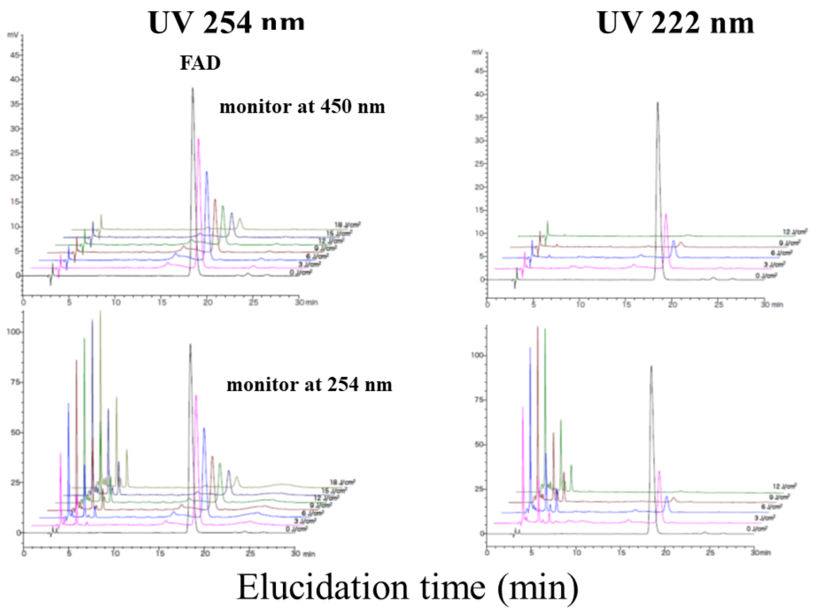

Fig. S6 HPLC elucidation profiles (up to 30 min) of FAD with increasing UV dose to 12 J/cm^2 and a step 2 J/cm^2

8. Absorption spectral change of purine nucleosides by irradiation at 222 and 254 nm

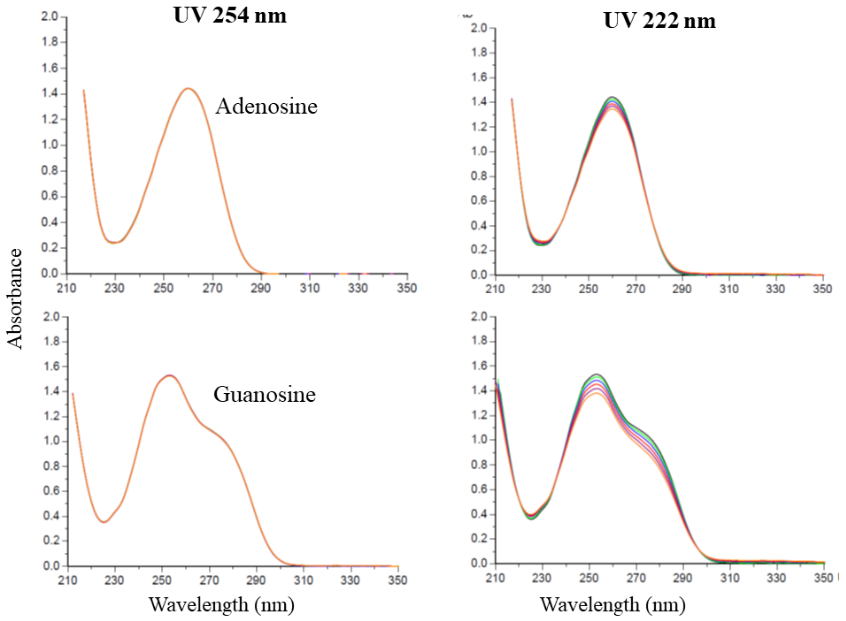

Fig. S7 Absorption spectrum changes of purine nucleosides with increasing doses at 222 and 254 nm from the black spectrum to the orange one (0−5.0 J/cm^2 with a step 1.0 J/cm^2). *aq.*solution 50 μM, optical path length = 10 mm

9. UV absorption spectra and HPLC elucidation profiles after UV photoirradiation of UpU

UpU in *aq*. solution (50 µg/mL) was irradiated at 222 and 254 nm. An example of the absorption spectral changes during photoirradiation is shown in Fig. S8. HPLC analyses in Fig. S9 were performed for the photoproducts and UpU with a 9:1 mixture solution of a TEAA buffer solution (0.1 M, pH = 7.0) and a TEAA (0.1 M) 50% / acetonitrile 50% solution. CPD and (6-4)PP were monitored at 215 nm and 313 nm, respectively. The spectrum of (6−4)PP was confirmed by the reference.

Reference: Franklin, W. A., Lo, K-M, Haseltine & W. A. Alkaline lability of fluorescent photoproducts produced in ultraviolet light-irradiated DNA. *J. Bio. Chem*. **257**, 13535–13543 (1982)

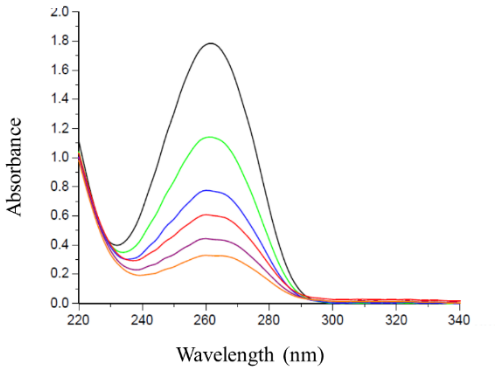

Fig. S8 Spectral change during 222-nm irradiation of UpU in *aq*. solution. Dose: 0, 0.9, 1.8, 2.7, 3.6 and 4.5 J/cm^2 from black to orange spectra

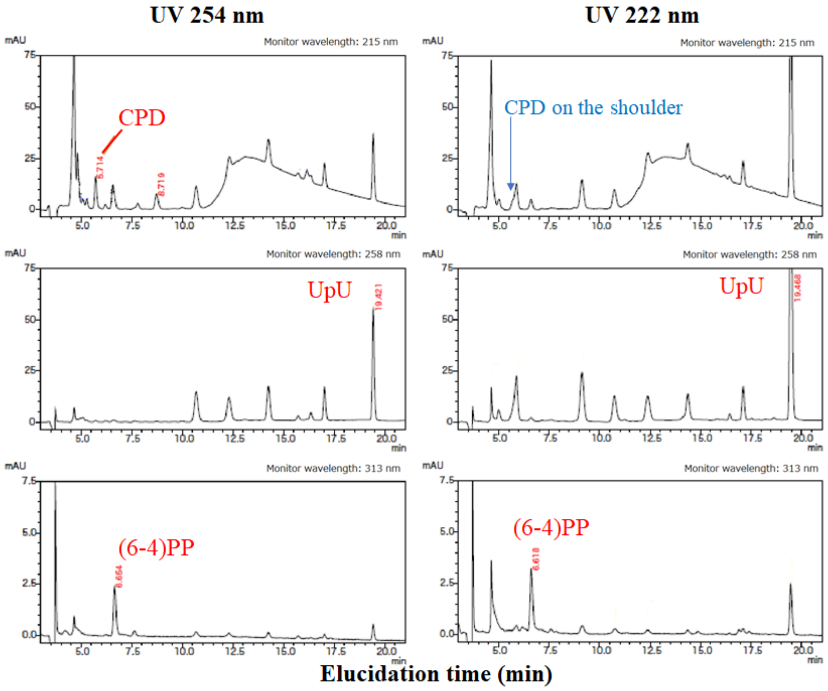

Fig. S9 HPLC elucidation profiles (up to 20 min) of photoproducts of UpU. The monitor wavelengths are (**upper**) 215 nm, (**middle**) 258 nm and (**lower**) 313 nm. Dose = 4.5 J/cm^2. At 254 nm, photoproducts include (6-4)PP and CPD, while at 222 nm products include (6-4)PP.

10. dTpdT after irradiation at 222 and 254 nm: HPLC elucidation profiles for the formation of CPD and (6-4)PP

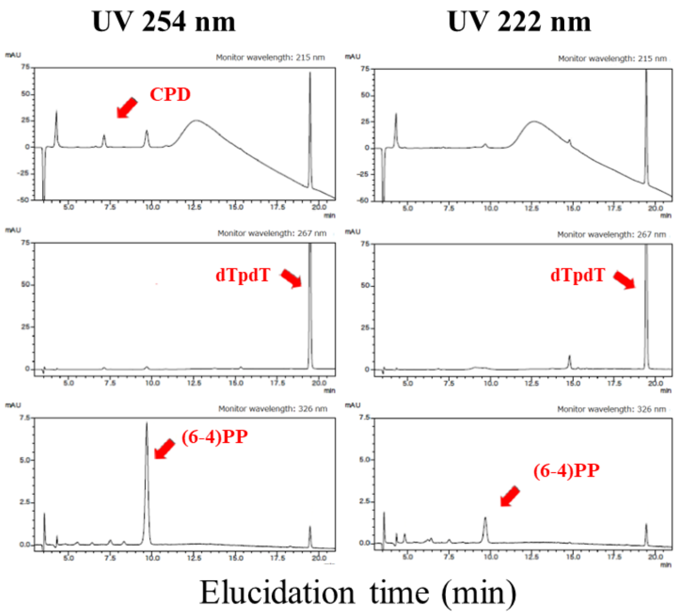

Fig. S10 HPLC elucidation profiles (up to 20 min) of photoproducts from dTpdT. (**left**) 254 nm, (**right**) 222 nm. The monitor wavelengths are (**upper**) 215 nm, (**middle**) 267 nm, (**lower**) 326 nm. Dose 7.2 J/cm^2, Irradiation time =120 min

11. UpU after irradiation at 254 nm: HPLC elucidation profile change by the self-reversion process during preservation under dark and room temperature conditions

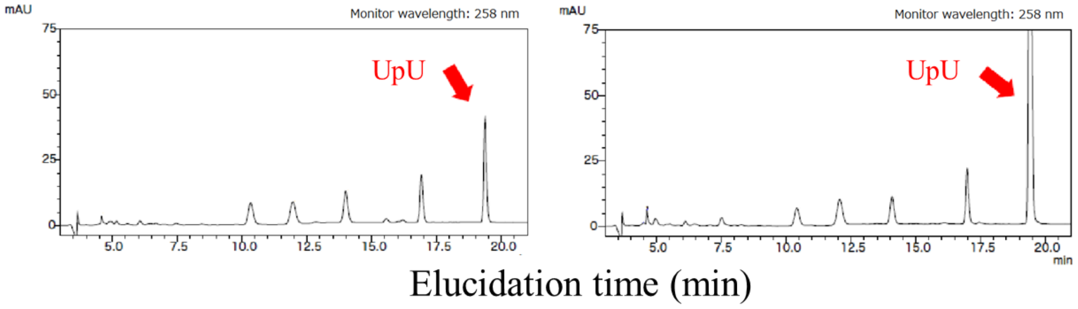

Fig. S11 HPLC elucidation profiles (up to 20 min) of photoproducts from UpU (**left)** immediately after 254-nm irradiation and (**right**) after eleven-day preservation under dark conditions at room temperature. The monitor wavelength is 258 nm. Dose = 3.6 J/cm^2, Irradiation time = 60 min.

12. Synthesis of RNA UpU

For the synthesis of RNA UpU, a levulinyl group was chosen for the transient protection of the 3’-

hydroxyl group. Figure S12 shows the synthesis path that proceeded quantitatively. The synthesis was successful because a levulinyl group was chosen for transient protection of the 3’−hydroxyl group. To confirm synthesis of UpU, product (10) in Fig.S12 was hydrolysed under alkaline conditions (0.5N NaOH) and two uridine monomers were measured using HPLC spectroscopy. Furthermore, synthesis products were analyzed by reversed-phase HPLC at monitoring wavelengths, 215, 258 and 313 nm, and using conventional absorption spectrometry at 220−340 nm. UpU in the TEAA buffer solution (pH = 7.0) and TEAA/acetonitrile (one-to-one mixture, pH = 7.0) had an absorption spectrum with a maximum at 259 nm, which was the same as that reported for uracil. Using the molar absorption coefficient of uracil, 10,100 /M cm, the concentration of the UpU solution was estimated to be 2.0 mg/cm^3. For UV irradiation purpose, the solution was diluted fortyfold with water. The absorption spectrum was measured using a spectrophotometer (Eppendorf Bio basic). HPLC analysis was performed with a 1:1 mixture of TEAA buffer solution and TEAA/acetonitrile.

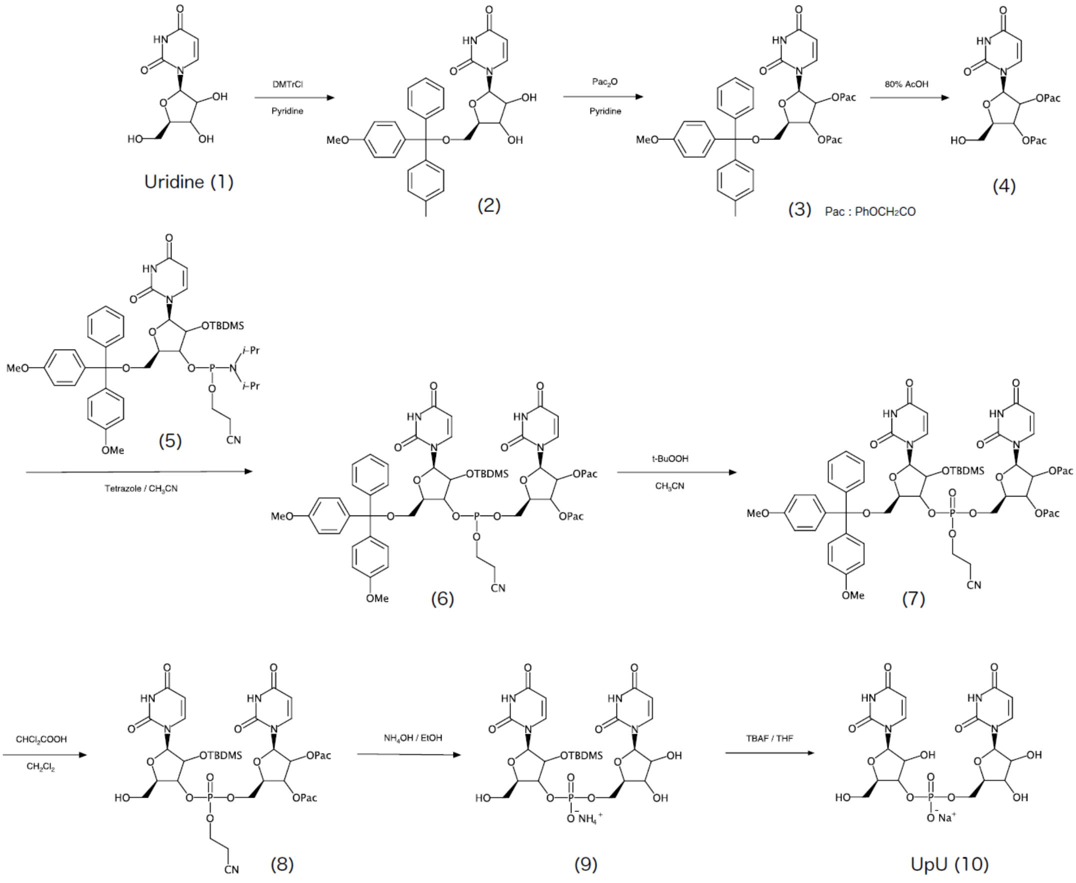

Fig. S12 Synthesis path for RNA UpU (10)

13. Photoabsorption of media solutions and culture plates for correction of effective UV flux

We examined the absorbance spectra of a phosphate-buffered saline (PBS) solution containing NaCl, KCl and sodium phosphate. The absorbance of PBS was lower than 0.01 mm^{-1} at 222 and 254 nm. PBS was used as a diluent to prepare the *E. coli* samples for UV irradiation. The light transmittance through the culture medium (3.1-mm thickness) that exhibited UV absorption due to proteins or amino acids was 69% for 222 nm and 63% for 254 nm. The effective power densities of the UV light sources used for inactivation were 92 $\mu W/cm^2$ for 222 nm and 83 $\mu W/cm^2$ for 254 nm. The culture media exhibited an absorption peak around 280 nm, which was due to proteins or amino acids in it. The effective dose at 365 nm was reduced to 81% using LB medium (10% diluted).
